# Association between Cooperative Attitude and High-Risk Behaviors on the Spread of COVID-19 Infection among Medical Students in Japan

**DOI:** 10.3390/ijerph192416578

**Published:** 2022-12-09

**Authors:** Chie Hirama, Zechen Zeng, Nobutoshi Nawa, Takeo Fujiwara

**Affiliations:** Department of Global Health Promotion, Tokyo Medical and Dental University, 1-5-45 Yushima, Bunkyo-ku, Tokyo 113-8510, Japan

**Keywords:** COVID-19, risk behaviors, university student, cooperative attitude, risk avoidance, trust

## Abstract

The impact of high-risk behaviors on the spread of COVID-19 infection among young people is an important problem to address. This study analyzed the association between cooperativeness and high-risk behaviors. We conducted a cross-sectional study among fourth-year medical students at Tokyo Medical and Dental University. The students were asked about cooperative attitude in a hypothetical situation of performing a task together with an unfamiliar classmate, who did not cooperate to complete the task previously. The response items were as follows: “cooperate”, “don’t want to cooperate and do it alone (non-cooperative)”, and “don’t want to cooperate and let the partner do it alone (punishment)”. Eating out and vaccine hesitancy were also treated as high-risk behaviors. Poisson regression was used to investigate the association between cooperative attitude and each high-risk behavior, adjusted for demographics. Of the 98 students, 23 (23.5%), 44 (44.9%), and 31 (31.6%) students chose “noncooperative”, “cooperative”, and “punishment”, respectively. Cooperative-type students exhibited 2.77-fold (PR: 2.77, 95% CI: 1.03–7.46), and punishment-type students exhibited 3.16-fold greater risk of eating or drinking out (PR: 3.16, 95% CI: 1.14–8.75) compared with those of the noncooperative type. Among medical students, the “cooperative” type and “punishment” type comprised the high-risk group for eating out during the pandemic.

## 1. Introduction

The COVID-19 pandemic has spread globally. In Japan, there have been more than 24,068,806 cases of infection and more than 48,642 deaths as of 25 November 2022 [1]. In addition, the spread of COVID-19 variants has been increasing, and controlling the spread is an urgent issue [2].

Due to Japan’s Constitution, the government cannot enforce strict policies to control citizens’ behavior to prevent the spread of COVID-19, such as wearing a mask or maintaining social distance [3,4,5,6]. The government can only encourage citizens to adopt these behavioral changes, known as “Avoid the three Cs (closed spaces, crowded spaces, and closed-contact settings)” [6]. Therefore, compliance with behavioral change is an issue because it varies greatly from person to person, especially among young people, who have a low risk of developing severe symptoms when infected with COVID-19 [7,8,9,10,11]. This strategy is called the “Japan model”, as opposed to the mandatory lockdowns based on laws or constitutional emergency clauses in other countries [12]. Under this model, the Japanese government calls on citizens to cooperate to prevent the spread of COVID-19 [13]. For example, the Japanese government asks people to wear masks or maintain social distance “to protect people important to you, such as family” [13]. In other words, it can be considered that public announcements would prompt people to become cooperative to prevent the spread of COVID-19. However, it is unclear whether cooperativeness is associated with behaviors related to avoiding infection by COVID-19, promoted by the Japanese government.

Human beings are a social species which relies on cooperation to survive and thrive. For example, in a hunter–gatherer lifestyle, sharing risk through cooperation is advantageous for survival, and humans receive more cooperation from others in raising children than other species [14,15]. However, cooperation may not always be beneficial for individuals. For example, when a tsunami strikes, fewer lives are likely to be lost if people put themselves first and escape instead of staying to help others [16]. This means that decisions on whether or not to cooperate should be made strategically based on the available information [17,18].

Whether people should cooperate during pandemics such as COVID-19 is unclear, because it may be similar to other unstable situations, e.g., a tsunami [16]. Previous studies have shown that during the COVID-19 pandemic, the number of related deaths is negatively associated with confidence in the government [19,20]. Consequently, a lack of trust in the government leads to low cooperation among people [21]. In contrast, COVID-19 deaths are positively related to social trust and group affiliations, leading to cooperation among people [22]. We considered the two types of cooperation: “vertical” [23] cooperation, which means cooperating with people in power, such as the government; and “horizontal” [23] cooperation, which means cooperating with peers, such as classmates in a university. We hypothesized that horizontal cooperation could negatively affect behavioral change during the pandemic because horizontal cooperation could come from either valuing human relationships or egalitarianism [24]. A previous study showed that horizontal bonding might involve excessive demands on group members, restrictions of freedom, excluding out-group members, and the “down-leveling” of norms [25]. These phenomena are considered the dark side of social capital [26]. Previous studies also confirmed that social capital could increase the risk of infection during the pandemic [27,28].

People who cooperate due to valuing human relationships would do so to maintain associations with others, whereas people who cooperate due to valuing egalitarianism may punish free riders. Thus, cooperation aims to develop the collective good and often requires a sacrifice of personal interests [29], whereas free riders want to receive benefits from the cooperative group. Free riders are more likely to be found in larger groups [30] and in repeated situations in which free riders learn the benefits [31]. The COVID-19 pandemic has spread all over the world, and states of emergency have repeatedly been declared; therefore, the two situations described are applicable. To prevent free riders, people in a group develop informal controls, including punishment [32,33,34,35]. Thus, if people punish disloyal others who have broken a promise to cooperate, such people can be considered for “punishment”, and are expected to punish free riders because they do not want free riders to receive benefits from systems of cooperation [32,33,34,35].

Thus, the attitude for horizontal cooperation (“cooperative attitude”) can affect human behavior. We classified the cooperative attitude into three types: “cooperative”, those who value horizontal cooperation; “noncooperative”, those who do not value it; and “punishment”, those who want to punish free riders. By identifying whether people are “cooperative”, “noncooperative”, or “punishment”, we examined the association between cooperative attitudes and changing behaviors during the pandemic. This information is valuable to encourage people to effectively change their behavior during future pandemics. However, because knowledge is essential for people’s safety behaviors [36,37], the knowledge level of participants needs to be similar. Thus, medical students were deemed to be optimal participants to investigate this hypothesis. In this study, we examined the association between cooperative attitude and the frequency of eating out and vaccine hesitancy among medical students in Japan.

## 2. Method

### 2.1. Study Participants

This study was conducted in 2021 on fourth-year medical students at Tokyo Medical and Dental University. The survey involved all fourth-year medical students (*N* = 113) who answered the questionnaire using Google Forms. There were 98 valid responses with informed consent to enroll (response rate 86.7%).

### 2.2. Measures

#### 2.2.1. Cooperative Attitude

First, the students were asked to imagine the following situation:

“*In a public health class, the professor has instructed you to work on two tasks with a partner. Your partner was randomly determined. You will not receive any credits unless you complete both tasks by the deadline. The first task is to be completed in two weeks, while the second is in a month. For both tasks, you are required to work with the same partner. Your partner is an unfamiliar classmate. For the first task, you and your partner agreed to share the first task and promised to finish it by the day before the deadline. However, at the agreed completion date, your partner told you via text messaging that the first assigned task had not been completed. You tried to ask for a reason but did not receive any answers. As such, you had to rush to finish the first task by yourself.*”

Then, the cooperative attitude was asked, to determine whether the student would like to choose “cooperate” for the second task even after the experience of being betrayed. The response items were as follows: “cooperate”, “don’t want to cooperate and do it alone (non-cooperative)”, and “don’t want to cooperate and let the partner do it alone (punishment)”.

#### 2.2.2. Risky Behaviors for the Spread of COVID-19 Infection

The frequency of eating or drinking out was asked using the following questions: “On average, how often did you eat out in the past six months?” and “On average, how often did you drink out in the past six months?” The response was chosen from five choices: 1 = “almost every day”, 2 = “three or four times a week”, 3 = “once or twice a week”, 4 = “several times a month”, and 5 = “almost never”. In this study, students who ate out once or twice a week on average or drank at a public establishment several times a month were considered to be “high-risk”.

#### 2.2.3. Vaccine Hesitancy

Vaccine hesitancy was asked using the following question: ‘‘Do you want to get vaccinated against COVID-19?” The response was from one of the three choices: 1 = “I want”, 2 = “I don’t want”, 3 = “I can’t answer”. Students who chose options 2 or 3 were defined as exhibiting “vaccine hesitancy”.

#### 2.2.4. Covariates

Covariates included gender (men or women), age (students who entered the university immediately after graduating from high school (approximately 22 years old), students who had gap years after graduating from high school (approximately 23 years old), and students who has graduated from another university (approximately 26 years old)). We did not ask for their age directly because some students were relatively more senior and may not want to be identified. The covariates also included parents’ income (<JPY 2 million, JPY 2 million ≤ JPY 4 million, JPY 4 million ≤ JPY 6 million, JPY 6 million ≤ JPY 8 million, JPY 8 ≤ JPY 10 million, ≥JPY10 million, “don’t know”, and “don’t want to answer”), birthplace (Greater Tokyo or not), living with family or not, and a history of mental disorders.

Additionally, to measure social engagement within the university, club activities (“joined sports club”, “joined cultural clubs”, and “did not join any clubs”), the number of friends in class, and the number of unfamiliar classmates (measured by the following response items: 0, 1, 2, 3–5, 6–10, 11–15, 16–20, 21–25, and ≥26), were asked. In addition, as for pre-university social engagement, the frequency of meeting high school friends in a year (measured by the following response items: 0, 1, 2, 3, 4, ≥5), the number of high school friends whom they meet after graduation (measured by the following response items: 0, 1, 2, 3–5, 6–10, 11–15, 16–20, 21–25, ≥26), having the experience of being betrayed by friends or teachers or not, and having favorite teachers during high school were asked.

Risk preference was asked in the form of “Do you consider yourself a person who would take risks in general?”, a question validated to present solid test–retest stability [38,39]. We adopted the two-choice form of Eysenck [40] to avoid participants centralizing in the middle.

### 2.3. Analysis

First, the association between demographic characteristics and cooperative attitudes was examined using the chi-squared test. Second, the Poisson regression model was used to estimate the association between cooperative attitude and each risk behavior, adjusted for age and sex. All analyses were performed with the STATA SE statistical package, version 15 (StataCorp LP, 2017. College Station, TX, USA).

## 3. Results

Table 1 shows the characteristics of the sample population. Two-thirds of the participants had entered university immediately after graduating from high school (i.e., 21–22 years old), 18% took gap years after graduating from high school (i.e., 22–24 years old), and 14% of them had the experience of going to another university (averaged 26 years old). Two-thirds of the students were male. Half of the students indicated that their household income was more than JPY 10 million. Over 70% of the students were from the capital area and lived with their family members. Five students had mental disorders. Eighteen students had high-risk preferences (18.4%). For the factor to measure social engagement within the university, half of the students said that they had over 21 unfamiliar classmates in class and fewer than 5 friends. Almost 90% of them belonged to a club. Social engagement in high school was also measured. Half of the students had had fewer than four meetings with high school friends in the past year, and had fewer than five high school friends. Additionally, 80% of the students said that they had no experience of being betrayed in high school, whereas 70% of them were found to have favorite teachers.

Table 2 shows the association between demographic characteristics and cooperative attitude. There were 44 students (45%) in the “cooperative” group, 23 (23%) in the “non-cooperative” group, and 31 (32%) in the “punishment” group. The frequency of meeting with high school friends was significantly associated with a cooperative attitude. The group of participants who met high school friends fewer than four times was more likely to hesitate in sharing the task (*p* = 0.003). Other demographic characteristics and risk preferences showed similar distribution among the cooperative attitudes.

Table 3 shows the association between cooperative attitudes and eating out at least once a week or drinking at a restaurant or bar a few times a month, adjusted for age and sex. Students who chose the “cooperative” group had a 2.77-fold greater risk of eating or drinking out compared with the “noncooperative” group. (prevalence rate (PR): 2.77, 95% confidence interval (CI): 1.03–7.46). The “punishment” group had 3.16-fold greater risk of eating or drinking out compared with the “noncooperative” group. (PR: 3.16, 95% CI: 1.14–8.75).

Table 4 shows the analysis results for the association between cooperative attitudes and vaccine hesitancy. No significant association was found; the “cooperative” group showed a similar risk of vaccine hesitancy in comparison with the “noncooperative” group (PR: 1.00, 95% CI: 0.56–1.80), as did the “punishment” group (PR: 0.94, 95% CI: 0.50–1.78).

## 4. Discussion

In this study, both the “cooperative” and “punishment” groups were positively associated with eating or drinking out compared with the “noncooperative” group. The results were consistent with our hypothesis that a cooperative attitude was positively related to risk behaviors. However, there was no significant association between cooperative attitude and vaccine hesitancy.

Previous studies have shown that young people are more likely to exhibit risk behaviors during the pandemic [10,41,42]. In particular, those who perceived their susceptibility to severe COVID-19 as being low were more likely to take risks to achieve social and emotional stimulation (such as in social gatherings) [10,43]. Although the importance of health communication to young people has been highlighted for behavioral changes to prevent a pandemic [10], the motivation for young people to cooperate has been less emphasized [10,44]. In this study, we focused on cooperative attitude as a motivation and measured the association between cooperative attitude and risk behaviors. To the best of our knowledge, this is the first report on the association between cooperative attitudes and risk behaviors during the COVID-19 pandemic among medical students.

The “cooperative” group was more likely to be agreeable and prosocial [45,46]. The preference for contributing to the group was generally working in tackling obstacles for the group [47]; however, it sometimes worked for group members to take dangerous actions, known as the dark side of social capital [26]. For example, the “cooperative” group may be susceptible to those who exhibit high-risk behaviors in the same group because behavioral contagion is likely to occur, i.e., susceptibility to false information and not maintaining social distancing during the pandemic [19] or promoting harmful drinking [48]. Thus, the “cooperative” group might continue to eat or drink out even during the pandemic. On the other hand, the “noncooperative” group values risk avoidance in specific situations, such as in a life-threatening scenario [16]. Thus, the benefit of cooperation has not been clear during the COVID-19 pandemic because it may be similar to a crisis such as a tsunami, in which noncooperative, individual behavior is recommended [16,17]. This is because noncooperation is more valuable than the collective good, which often requires the sacrifice of personal interests [29]. Thus, these persons might choose not to go out to reduce their risk of infection.

The “punishment” group punishes free riders because they do not want free riders to benefit from the system of cooperation [32,33,34,35]. However, in situations where they cannot punish free riders, such as someone eating or drinking out during the pandemic, the “punishment” group may want the same benefits as free riders to maintain an egalitarian society [49]. Further qualitative research is needed to understand why the people in the “punishment” group continued to eat or drink out even during the pandemic.

We did not find a significant association between cooperative attitudes and vaccine hesitancy. Previous studies showed that health literacy was positively associated with vaccination [50,51]. Additionally, highly educated groups were found to be more likely to have an intention to be vaccinated [52]. In this study, all participants were medical students in the same university with a similar level of healthcare literacy. Thus, most of them would have intended to be vaccinated regardless of their cooperative attitude.

This study has several limitations. First, for the assessment of cooperative attitude, unvalidated measurements were used based on real situations in the university. Future studies incorporating different measures for cooperativeness may be warranted to replicate our findings. Second, cooperative attitudes were measured by the response to a hypothetical situation, which might be different from actual behaviors. Third, this study consisted of only fourth-year medical students at a single university. Thus, the result may not apply to all young people in Japan. Finally, we did not assess the reasons for cooperation and risk behaviors. Hence, our measure of cooperative attitude may be a proxy for other reasons for risk behaviors during the pandemic.

## 5. Conclusions

In conclusion, the cooperative attitude of young people was associated with risk behaviors during the COVID-19 pandemic. Those who value horizontal cooperation for either human relationships or egalitarianism exhibit a higher risk of eating or drinking out, even during the pandemic. Our study suggests that government public service announcements to young people could be more effective if they emphasize the importance of risk avoidance rather than demanding young people to be cooperative.

## Figures and Tables

**Table 1:** Characteristics of sample.

			Total (*n* = 98)	
			*n*	%
Demographics	Sex	Male	62	63.3
		Female	36	36.7
	Age	Have experience of going to another universities	14	14.3
		Have blank year after graduating universities	18	18.4
		Enter the university immediately after graduating universities	66	67.4
	Household income	<JPY 10 million	27	27.6
		≥JPY 10 million	45	45.9
		Don’t know/don’t want to answer	26	26.5
	Birthplace	Around Tokyo	71	72.5
		Other	27	27.6
	Living with family	Live with family	70	71.4
		Live alone	28	28.6
	Mental disorders	Yes	5	5.10
		No	93	94.9
	Risk preference	Yes	18	18.4
		No	80	81.6
Social capital in university	Number of familiar friends in the class	0~5	54	55.1
		6~10	27	27.6
		>11	17	17.3
	Number of unfamiliar friends in the class	0~10	18	18.4
		11~20	31	31.6
		>21	49	50.0
	Club activities	Belong to	86	87.8
		Do not belong to	12	12.2
Social capital in high school	Frequency of meeting high school friends	0~4	53	54.1
		≥5	45	45.9
	Number of high school friends who they meet now	0~5	49	50.0
		6~10	33	33.7
		>11	16	16.3
	Experience of being betrayed by someone	no	77	78.6
		Only by teachers	2	2.04
		Only by friends	11	11.2
		By both	8	8.16
	Have favorite teacher	Yes	70	71.4
		No	28	28.6

**Table 2:** Sharing tasks with an unfamiliar classmate (reference: cooperative).

		Noncooperative (23, 23.5%)		Cooperative (44, 44.9%)		Punishment (31, 31.6%)		*p*
		*n*	%	*n*	%	*n*	%	
Do you like to take risks in general?	No	20	87.0	33	75.0	27	87.1	0.31
	Yes	3	13.0	11	25.0	4	12.9	
How much do your parents earn?	<JPY 1000	9	39.1	12	27.3	6	19.4	0.42
	≥JPY 1000	8	34.8	19	43.2	18	58.1	
	No answer	6	26.1	13	29.6	7	22.6	
Are you from areas from Tokyo?	No	5	21.7	15	34.1	7	22.6	0.42
	Yes	18	78.3	29	65.9	24	77.4	
Are you living with your family now?	No	9	39.1	13	29.6	6	19.4	0.28
	Yes	14	60.9	31	70.5	25	80.7	
Have you ever suffered from mental disorders?	Yes	1	4.35	3	6.82	1	3.23	0.77
	No	22	95.7	41	93.2	30	96.8	
Do you belong to club activities?	Yes	21	91.3	39	88.6	26	83.9	0.42
	No	2	8.70	5	11.4	5	16.1	
How many familiar friends do you have in your class?	0~5	13	56.5	23	52.3	18	58.1	0.93
	6~10	7	30.4	13	30.0	7	22.6	
	>11	3	13.0	8	18.2	6	19.4	
How many unfamiliar friends do you have in your class?	0~10	6	26.1	8	18.2	4	12.9	0.26
	11~20	4	17.4	18	40.9	9	29.0	
	>21	13	56.5	18	40.9	18	58.1	
How often have you met your high school friends in the past year?	0~4	19	82.6	23	52.3	11	35.5	**0.003**
	≥5	4	17.4	21	47.7	20	64.5	
How many high school friends do you have who you meet after graduation?	0~5	14	60.9	21	47.7	14	45.2	0.75
	6~10	7	30.4	15	34.1	11	35.5	
	>11	2	8.70	8	18.2	6	19.4	
Have you been betrayed by your friends or teachers?	No	16	69.6	34	77.3	27	87.1	0.16
	Only by teachers	1	4.35	0	0	2	3.23	
	Only by friends	2	8.70	8	18.2	11	3.23	
	By both	4	17.4	2	4.55	8	6.45	
Did you have favorite teachers in your school?	No	11	47.8	9	20.5	8	25.8	0.057
	Yes	12	52.2	35	79.6	23	71.4	

Bold indicates *p* < 0.05.

**Table 3:** Association between sharing tasks with an unknown classmate and eating out at least once a week or drinking at a restaurant several times a month.

		Prevalence of Eating Out	Crude		Model 1 (Sex, Age)	
		*N*, %	PR	95% Cl	PR	95% Cl
Sharing tasks with an unfamiliar classmate	Noncooperative	5, 21.7%	Reference		Reference	
	Cooperative	23, 52.3%	2.40	0.91–6.32	**2.77**	**1.03–7.46**
	Cooperative and punishment	17, 54.8%	2.52	0.93–6.84	**3.16**	**1.14–8.75**

Bold indicates *p* < 0.05.

**Table 4:** Association between sharing tasks with an unknown classmate or a familiar classmate and COVID-19 vaccination hesitancy.

		Prevalence of Vaccine Hesitancy	Crude		Model 1 (Sex, Age)	
		*N*, %	PR	95% Cl	PR	95% Cl
Sharing tasks with an unfamiliar classmate	Noncooperative	5, 21.7%	Reference		Reference	
	Cooperative	9, 20.5%	1.02	0.58–1.79	1.00	0.56–1.80
	Cooperative and punishment	8, 25.8%	0.95	0.51–1.76	0.94	0.50–1.78

## Data Availability

This study is a secondary data analysis of survey data as part of a class.
